# Supplementary material for: Fluorescent SiO2@Tb3+(PET-TEG)3Phen Hybrids as Nucleating Additive for Enhancement of Crystallinity of PET
Source: Polymers (Basel). 2020 Mar 4;12(3):568. doi: 10.3390/polym12030568 (PMC7182918; doi:10.3390/polym12030568)
Supplement: Supplementary file 1 [file polymers-12-00568-s001.pdf]

Supplementary information

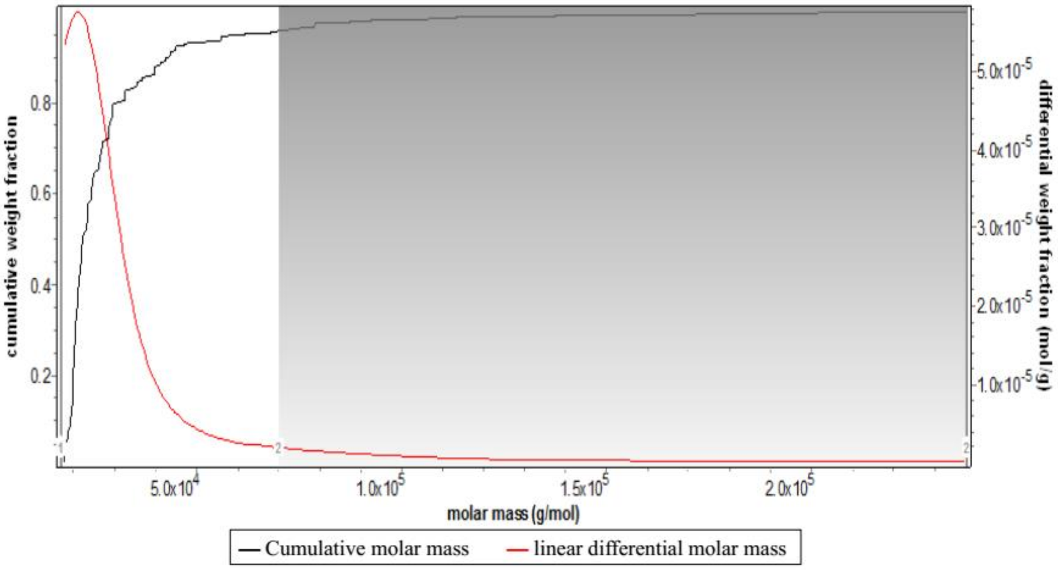

Figure S1. The distribution analysis of molecular weight of PET-TEG

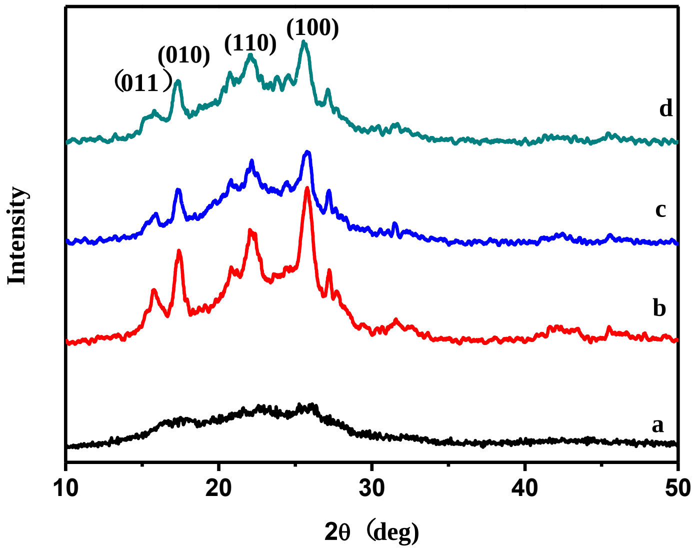

Figure S2. The XRD spectra of PET containing different content of $SiO_2@Tb^{3+}(PET\text{-}TEG)_3Phen$ a: 0%, b: 1%, c: 2%, d: 3%

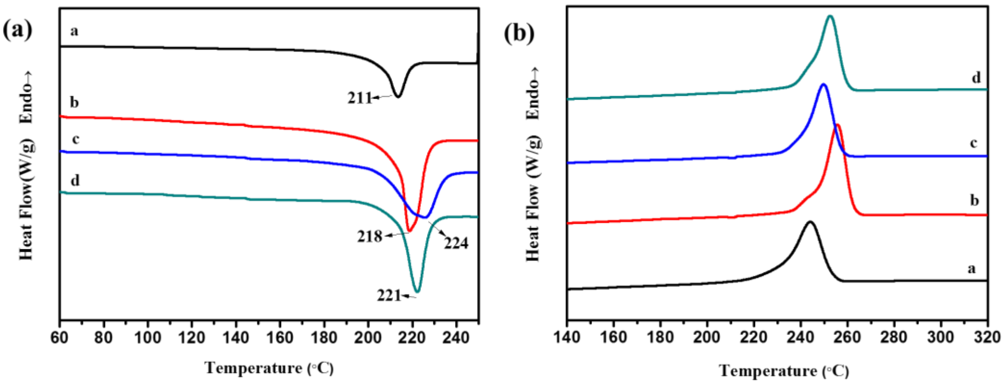

Figure S3. The DSC cooling Tc(a) and heating Tm(b) curves of different content of SiO_2@Tb^{3+}(PET-TEG)$_3$Phen a: 0%, b: 1%, c: 2%, d: 3%;

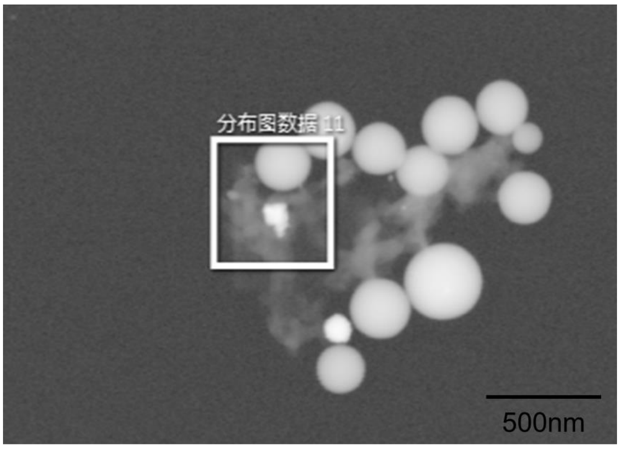

Figure S4. The SEM-EDS image of SiO_2@Tb^{3+}(PET-TEG)$_3$ Phen

Table S1 Thermodynamic performance of composites

W(%)	Tc(℃)	Tm(℃)	△Hc(J/g)	△Hm(J/g)	Xc(%)
0	211.15	244.32	44.38	43.01	30.72
1	218.49	255.93	45.02	44.23	31.93
2	224.01	249.86	45.73	44.87	32.70
3	221.24	252.47	45.36	43.89	32.32
